# Complete Chloroplast Genome of an Endophytic *Ostreobium* sp. (Ostreobiaceae) from the U.S. Virgin Islands

**DOI:** 10.1128/mra.00272-23

**Published:** 2023-04-24

**Authors:** Mustafa Alesmail, Yulissa Becerra, Kimberly J. Betancourt, Shelly M. Bracy, Anevay T. Castro, Cynthia Cea, Justin Chavez, Janet Del Angel, Edgar Diaz, Yael Diaz-Guzman, Jonathan Dominguez, Jocelynnicole G. Estrada, Lashabelle G. Frei, Paul W. Gabrielson, Andrea Gallardo, Miriam R. Garcia, Eva Gonzalez, Anthony Gonzalez Rocha, Diego Guzman-Bermudez, Cassidy R. Hebert, Marlene Hernandez, Jeffery R. Hughey, Zachary Lee, Alexandra Leyva Romero, Eric Martinez, Nathaniel Martinez, Kazimiera H. Medina, Miguel Morales, Alexis M. Moreno, Isabella Nava, Alyssa N. Nono, Samuel A. Ochoa, Amy Perez, Natasha Perez, Edwin Perez Pulido, Sophie Poduska, Kimberly N. Ramirez, Denise Reyes, Kelsey Richardson, Juanaisa Rodriguez, Alondra M. Rodriguez, Clarisa Serrano-Lopez, Andrea G. Velasquez, Gezelle Villanueva

**Affiliations:** a Division of Mathematics, Science, and Engineering, Hartnell College, Salinas, California, USA; b Biology Department and Herbarium, University of North Carolina at Chapel Hill, Chapel Hill, North Carolina, USA; Queens College Department of Biology

## Abstract

We present the complete chloroplast genome sequence of an endophytic *Ostreobium* sp. isolated from a 19th-century coralline red algal specimen from St. Croix, U.S. Virgin Islands. The chloroplast genome is 84,848 bp in length, contains 114 genes, and has a high level of gene synteny to other Ostreobiaceae.

## ANNOUNCEMENT

*Ostreobium* Bornet & Flahault is a siphonous marine green algal symbiont with three currently recognized species (1–3). *Ostreobium* plays an important role in decalcification and providing photosynthates to the corals, especially during bleaching events (4). *Ostreobium* also occurs as an endophyte in various crustose coralline algae (5, 6). Twelve complete *Ostreobium* chloroplast genomes isolated from corals have been sequenced (1, 3, 7–9). We characterized the complete chloroplast genome of an *Ostreobium* coralline endophyte to contribute to the systematics and bioinformatics of Ostreobiaceae.

The specimen analyzed here was a 3- by 3-mm coralline fragment of Hydrolithon boergesenii (Foslie) Foslie collected in 1892 by Frederik Børgesen from St. Croix, U.S. Virgin Islands, and housed at ambient temperature in the Norwegian University of Science and Technology Herbarium under voucher number TRH A14-720. The fragment was ground with a mortar and pestle, and DNA was extracted using the DNeasy Blood and Tissue kit (Qiagen) following the manufacturer’s protocol with two modifications: the binding step was 4,000 × *g* for 3 min and the DNA was eluted in 40 μL Tris-acetate-EDTA (TAE) after 7 min of incubation (10). The 150-bp paired-end library was constructed with the KAPA HyperPlus kit (Roche) and sequenced on an Illumina NovaSeq 6000. The analysis generated 17,620,232 reads. The reads were filtered using the default BBDuk settings in Geneious Prime 2019.1.3 (Biomatters Limited). The chloroplast genome was assembled *de novo* using the filtered reads with the default settings in MEGAHIT 1.2.9 (11). The assembly yielded 62,269 small contigs with an *N*_50_ of 561 and GC content of 50.2%. A single *Ostreobium* sp. chloroplast contig with 249× coverage was identified by Nucleotide BLAST search. The final complete chloroplast genome was circularized by removing the overlapping ends, manually adjusting the start position to Ostreobium quekettii Bornet & Flahault voucher CS-1386 (GenBank accession number OK189528), and confirming its accuracy with the map-to-reference function using default settings in Geneious Prime. The annotation was performed using the default settings in GeSeq 2.03 (12), followed by manual corrections of gene start and stop positions according to NCBI ORFfinder and Sequin 15.5 (13). Nucleotide identities were calculated by BLAST search using the more-dissimilar-sequence optimization setting.

**FIG 1:**
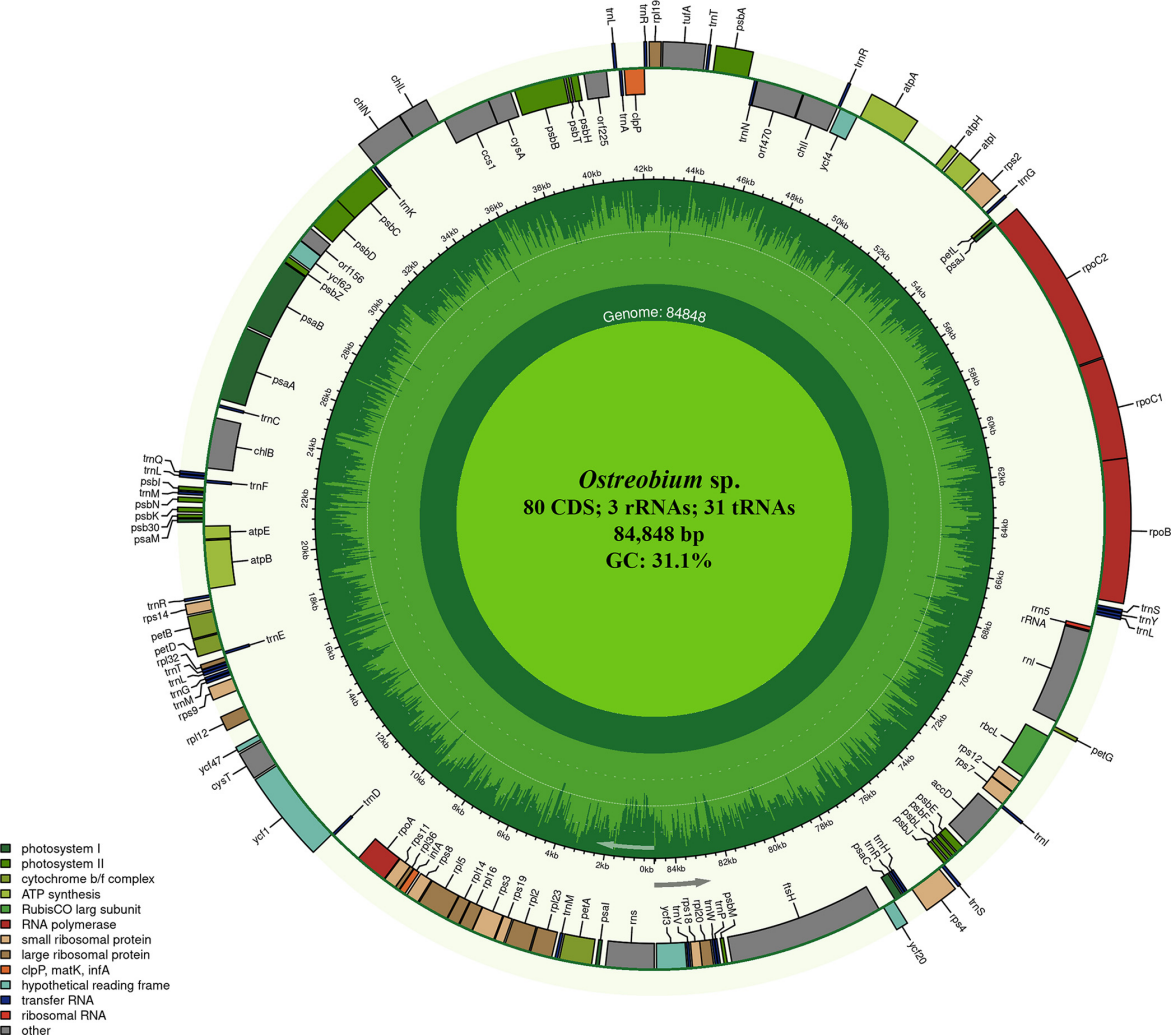
Complete chloroplast genome of *Ostreobium* sp. The genome was annotated using GeSeq (12), NCBI ORFfinder, and Sequin 15.5 (13), and mapped with Chloroplot 0.2.4 (14). The center contains genome characteristics. The innermost ring shows the genome length. The second ring displays the GC content and direction of transcription, as indicated by the two arrows. The final ring shows the genes. Genes transcribed clockwise are on the inside; counterclockwise transcriptions are on the outside. The color coding corresponds to genes of different groups as listed in the key in the bottom left. CDS, coding sequences.

The complete circular chloroplast genome of the *Ostreobium* sp. is 84,848 bp in length and has a GC content of 31.1% (Fig. 1). The genome contains 114 genes including 80 protein-coding, 31 tRNA, and 3 rRNA genes. Seven of the genes contain one intron (*ccs1*, *rpl5*, *rpl23*, *rpoB*, *rpoC1*, *rps4*, and *ycf3*). Gene content and organization are nearly identical to those of *O. quekettii* CS-1386 isolated from coral from the Great Barrier Reef. The St. Croix *Ostreobium* sp. chloroplast genome, however, lacked an ATP synthase CF0 subunit I gene start codon. The chloroplast genome of *Ostreobium* sp. has a 75.26% to 80.57% nucleotide identity to 10 *O. quekettii* and two *Ostreobium* species sequences deposited in GenBank. This study shows that DNA from *Ostreobium* can be retrieved from museum specimens and advances the understanding of *Ostreobium* genomics, biogeography, and systematics.

### Data availability.

The complete chloroplast genome sequence of *Ostreobium* sp. is available in GenBank under accession number OQ349515. The associated BioProject, SRA, and BioSample numbers are PRJNA948419, SRS17145397, and SAMN33902323, respectively. The chloroplast genome referenced in the text was *Ostreobium quekettii* GenBank accession number OK189528.

## References

[1] Verbruggen H, Marcelino VR, Guiry MD, Cremen MCM, Jackson CJ. 2017. Phylogenetic position of the coral symbiont Ostreobium (Ulvophyceae) inferred from chloroplast genome data. J Phycol 53:790–803. doi:10.1111/jpy.12540. 28394415

[2] Massé A, Domart-Coulon I, Golubic S, Duché D, Tribollet A. 2018. Early skeletal colonization of the coral holobiont by the microboring Ulvophyceae Ostreobium sp. Sci Rep 8:2293. doi:10.1038/s41598-018-20196-5. 29396559 PMC5797222

[3] Iha C, Dougan KE, Varela JA, Avila V, Jackson CJ, Bogaert KA, Chen Y, Judd LM, Wick R, Holt KE, Pasella MM, Ricci F, Repetti SI, Medina M, Marcelino VR, Chan CX, Verbruggen H. 2021. Genomic adaptations to an endolithic lifestyle in the coral-associated alga Ostreobium. Curr Biol 31:1393–1402.e5. doi:10.1016/j.cub.2021.01.018. 33548192

[4] Fine M, Loya Y. 2002. Endolithic algae: an alternative source of photoassimilates during coral bleaching. Proc Biol Sci 269:1205–1210. doi:10.1098/rspb.2002.1983. 12065035 PMC1691023

[5] Steveninck ED, Breeman AM. 1981. Biomass and relative coverage of benthic algae in the fore-reef of Curaçao (Netherlands Antilles) in relation to production. Mar Ecol Prog Ser 6:257–265. doi:10.3354/meps006257.

[6] Tribollet A, Payri C. 2001. Bioerosion of the coralline alga Hydrolithon onkodes by microborers in the coral reefs of Moorea, French Polynesia. Oceanol Acta 24:329–342. doi:10.1016/S0399-1784(01)01150-1.

[7] Marcelino VR, Verbruggen H. 2016. Multi-marker metabarcoding of coral skeletons reveals a rich microbiome and diverse evolutionary origins of endolithic algae. Sci Rep 6:31508. doi:10.1038/srep31508. 27545322 PMC4992875

[8] Pasella MM, Lee M-FE, Marcelino VR, Willis A, Verbruggen H. 2022. Ten Ostreobium (Ulvophyceae) strains from Great Barrier Reef corals as a resource for algal endolith biology and genomics. Phycologia 61:452–458. doi:10.1080/00318884.2022.2064132.

[9] Del Campo J, Pombert JF, Šlapeta J, Larkum A, Keeling PJ. 2017. The ‘other’ coral symbiont: Ostreobium diversity and distribution. ISME J 11:296–299. doi:10.1038/ismej.2016.101. 27420029 PMC5315466

[10] Garcia AN, Ramos JH, Mendoza AG, Muhrram A, Vidauri JM, Hughey JR, Hartnell College Genomics Group. 2022. Complete chloroplast genome of topotype material of the coast live oak Quercus agrifolia née var. agrifolia (Fagaceae) from California. Microbiol Resour Announc 11:e0000422. doi:10.1128/mra.00004-22. 35254126 PMC9022551

[11] Li D, Liu CM, Luo R, Sadakane K, Lam TW. 2015. MEGAHIT: an ultra-fast single-node solution for large and complex metagenomics assembly via succinct de Bruijn graph. Bioinformatics 31:1674–1676. doi:10.1093/bioinformatics/btv033. 25609793

[12] Tillich M, Lehwark P, Pellizzer T, Ulbricht-Jones ES, Fischer A, Bock R, Greiner S. 2017. GeSeq – versatile and accurate annotation of organelle genomes. Nucleic Acids Res 45:W6–W11. doi:10.1093/nar/gkx391. 28486635 PMC5570176

[13] Benson DA, Cavanaugh M, Clark K, Karsch-Mizrachi I, Ostell J, Pruitt KD, Sayers EW. 2018. GenBank. Nucleic Acids Res 46:D41–D47. doi:10.1093/nar/gkx1094. 29140468 PMC5753231

[14] Zheng S, Poczai P, Hyvönen J, Tang J, Amiryousefi A. 2020. Chloroplot: an online program for the versatile plotting of organelle genomes. Front Genet 11:576124. doi:10.3389/fgene.2020.576124. 33101394 PMC7545089

